# Data on recovery of 21 amino acids, 9 biogenic amines and ammonium ions after spiking four different beers with five concentrations of these analytes

**DOI:** 10.1016/j.dib.2016.09.011

**Published:** 2016-09-15

**Authors:** Begoña Redruello, Victor Ladero, Beatriz del Rio, María Fernández, M. Cruz Martín, Miguel A. Alvarez

**Affiliations:** Dairy Research Institute (IPLA-CSIC), Paseo Río Linares s/n, 33300 Villaviciosa, Asturias, Spain.

**Keywords:** Biogenic amines, Amino acids, Beer, Recovery

## Abstract

A novel chromatographic method for the simultaneous analysis of nine biogenic amines, 21 amino acids and ammonium ions in beer has been recently described in “A UHPLC method for the simultaneous analysis of biogenic amines, amino acids and ammonium ions in beer” (Redruello et al., 2017) [1]. The present article provides recovery data of the 31 analytes after spiking four different beers with five concentrations of each analyte (15, 30, 60, 120 and 240 µM).

**Specifications Table**

**Table:** 

Subject area	*Chemistry*
More specific subject area	*Food Chemistry*
Type of data	*Table*
How data was acquired	*Ultra high-performance liquid chromatography (UHPLC). Model: H-Class Acquity UPLC*^*TM*^*system (*Waters, Milford, MA, USA)
Data format	*Analyzed*
Experimental factors	*Four beer samples of different matrix complexity (an alcohol-free french lager, an artisan spanish lager, and two abbey-style dark belgian ale beers) and a 0.1 N HCl solvent solution were used as matrices in this work. Analytes’ mixtures containing 9 biogenic amines, 21 amino acids, and ammonium ions at five different concentrations (15, 30, 60, 120 and 240 μM) were added (spiked) to each of the five matrices.*
Experimental features	*100 µL of each sample were derivatized with diethylethoxymethylenmalonate (DEEMM) according to*[2]*, filtered through a 0.2 µm PTFE membrane (VWR, Barcelona, Spain) and one microliter injected into the chromatographic system. Data were analyzed with Empower 2.0 software (Waters).*
Data source location	*Breweries in Spain, France and Belgium.*
Data accessibility	*Data is with this article*

**Value of the data**

• These dataset allow researchers to evaluate the accuracy of the method developed to simultaneously quantify biogenic amines, amino acids and ammonium ions in different beers [1].
• Some biogenic amines are toxic, especially to susceptible individuals [3], [4], [5], [6]. Thus, these data are useful to researchers involved in beer safety and beer quality research projects, with a particular interest in evaluating the content of biogenic amines and their precursor amino acids in this beverage.

## 1 Data

Recovery data of 9 biogenic amines, 21 amino acids, and ammonium ions after spiking four different beers with five concentrations of a standard mixture containing these 31 analytes (15, 30, 60, 120 and 240 µM) are presented in Table 1.

## 2 Experimental design, materials and methods

Four beer samples of different matrix complexity (an alcohol-free french lager, an artisan spanish lager, and two abbey-style dark belgian ale beers) and a 0.1 N HCl solvent solution were used as matrices in this work. Analytes’ mixtures containing 9 biogenic amines, 21 amino acids, and ammonium ions at five different concentrations (15, 30, 60, 120 and 240 μM) were added (spiked) to each of the five matrices. One hundred microliters of each sample were derivatized with DEEMM, further separated by UHPLC and peak areas determined, as described in [2]. Recovery of each analyte was calculated as [(peak area measured in the spiked sample)–(peak area measured in the non-spiked sample)/(area measured in the solvent 0.1 N HCl solution]×100. Mean recovery for each analyte and each spiked concentration was calculated from the individual recovery data of the five matrices used in the experiment (see Table 1).

## Figures and Tables

**Table 1:** Mean recovery values (%) of each analyte at each spiked concentration (µM) from individual recoveries in four spiked beer samples. The results for biogenic amines are highlighted in grey.

Compound	15 μM	30 μM	60 μM	120 μM	240 μM
Aspartic acid	87.97	97.15	99.43	100.91	98.41
Glutamic acid	94.25	99.27	96.57	99.04	98.15
Asparagine	100.05	100.74	102.59	102.12	97.98
Serine	102.81	102.44	104.27	103.29	99.39
Glutamine	117.72	100.44	97.72	96.69	97.32
Histidine	90.22	101.87	102.90	103.61	97.55
Glycine	94.13	97.79	102.81	101.16	98.91
Threonine	97.48	98.13	103.71	103.40	100.06
Arginine	90.64	94.16	105.14	105.81	100.85
GABA	96.07	100.21	96.35	101.77	105.67
Alanine	95.01	111.38	109.11	110.34	102.92
Proline	103.61	86.98	94.82	96.39	98.12
Ammonium ion	64.52	76.27	86.17	101.63	96.39
Ethanolamine	91.70	102.57	102.01	102.89	100.30
Tyrosine	101.05	101.96	103.90	101.59	105.53
Agmatine	91.84	109.10	106.59	91.93	89.87
Histamine	108.75	103.11	102.01	100.48	98.17
Valine	96.36	99.85	102.03	101.17	98.51
Methionine	85.39	87.75	89.91	86.41	86.13
Tryptophan	100.78	99.02	100.99	98.39	96.30
Isoleucine	98.82	103.56	104.06	102.86	99.65
Leucine	95.30	100.71	103.69	104.51	98.91
Phenylalanine	94.73	98.53	101.72	102.48	99.33
Ornithine	102.03	104.38	103.03	100.71	98.75
Lysine	97.73	103.18	103.39	102.72	99.53
Ethylamine	113.33	94.89	98.12	101.05	101.91
Tyramine	104.77	108.32	103.13	101.42	99.51
Putrescine	104.59	99.47	105.75	103.30	100.57
Tryptamine	98.38	102.89	107.10	99.07	95.44
Cadaverine	108.89	108.34	107.35	103.89	101.01
Phenylethylamine	104.83	106.85	106.43	101.75	99.94
