# Supplementary material for: Data on recovery of 21 amino acids, 9 biogenic amines and ammonium ions after spiking four different beers with five concentrations of these analytes
Source: Data Brief. 2016 Sep 15;9:398–400. doi: 10.1016/j.dib.2016.09.011 (PMC5035235; doi:10.1016/j.dib.2016.09.011)
Supplement: Supplementary file 1 — Supplementary material [file mmc1.doc]

# Villaviciosa, August 29, 2026

Conflict of interest Form_DIB-D-16-00634

Dear Managing Editor Sarah O'Loughlin,

The contents of this manuscript are our original work which has not been published previously, in whole or in part, and is not under consideration in another journal simultaneously with our submission to Data in Brief. All authors have contributed significantly to the work and, additionally, have revised and approved the manuscript. All authors declare that the research was conducted in the absence of any commercial or financial relationships that could be construed as a potential conflict of interest.

Yours sincerely,


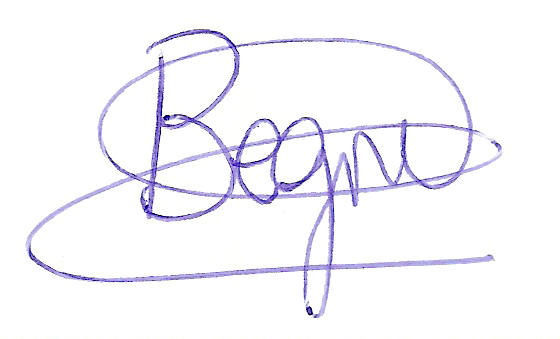


Begoña Redruello

Corresponding author of DIB-D-16-00634
